# Integrin α11β1 in tumor fibrosis: more than just another cancer-associated fibroblast biomarker?

**DOI:** 10.1007/s12079-022-00673-3

**Published:** 2022-04-04

**Authors:** Cédric Zeltz, Roya Navab, Ritva Heljasvaara, Marion Kusche-Gullberg, Ning Lu, Ming-Sound Tsao, Donald Gullberg

**Affiliations:** 1 Department of Biomedicine, Matrix Biology Group, Centre for Cancer Biomarkers, University of Bergen, Jonas Lies vei 91, 5009 Bergen, Norway; 2 Princess Margaret Cancer Center, University Health Network, Toronto, ON M5G 1L7 Canada; 3 Oulu Center for Cell-Matrix Research, Faculty of Biochemistry and Molecular Medicine, University of Oulu, Oulu, Finland; 4 Department of Laboratory Medicine and Pathobiology, University of Toronto, Toronto, ON M5G 1X8 Canada

**Keywords:** Tumor microenvironment, Cancer-associated fibroblast, Integrin, Fibrosis, Cancer therapy

## Abstract

There is currently an increased interest in understanding the role of the tumor microenvironment (TME) in tumor growth and progression. In this context the role of integrins in cancer-associated fibroblasts (CAFs) will need to be carefully re-evaluated. Fibroblast-derived cells are not only in the focus in tumors, but also in tissue fibrosis as well as in inflammatory conditions. The recent transcriptional profiling of what has been called “the pan-fibroblast cell lineage” in mouse and human tissues has identified novel transcriptional biomarker mRNAs encoding the secreted ECM proteins dermatopontin and collagen XV as well as the phosphatidylinositol-anchored membrane protein Pi16. Some of the genes identified in these fibroblasts scRNA-seq datasets will be useful for rigorous comparative characterizations of fibroblast-derived cell subpopulations. At the same time, it will be a challenge in the coming years to validate these transcriptional mRNA datasets at the protein-(expression) and at tissue-(distribution) levels and to find useful protein biomarker reagents that will facilitate fibroblast profiling at the cell level. In the current review we will focus on the role of the collagen-binding integrin α11β1 in CAFs, summarizing our own work as well as published datasets with information on α11 mRNA expression in selected tumors. Our experimental data suggest that α11β1 is more than just another biomarker and that it as a functional collagen receptor in the TME is playing a central role in regulating collagen assembly and matrix remodeling, which in turn impact tumor growth and metastasis.

## Introduction

**Fig. 1:**
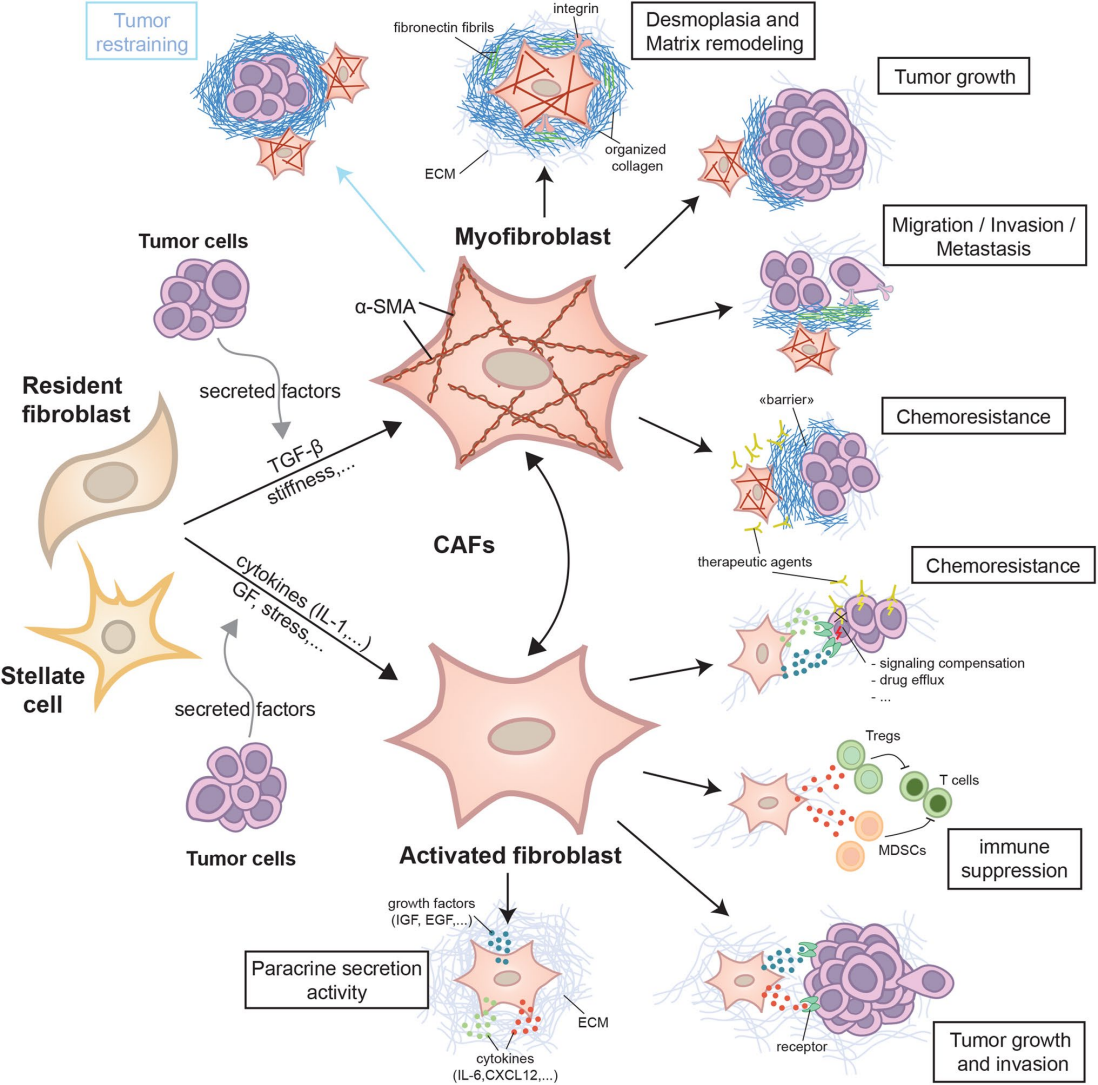
**Cancer-associated fibroblasts.** Cancer-associated fibroblasts (CAFs) are activated fibroblasts, which are called myofibroblasts when they harbor myofibroblastic features such as α-SMA expression. They are mainly derived from resident fibroblasts but can also be contributed from stellate cells in the context of liver and pancreatic cancer (as well as other cell types such as adipocytes and endothelial cells). Tumor cells by secreting soluble factors could initiate fibroblast activation and myofibroblast differentiation. Activated fibroblasts can differentiate into myofibroblasts and this process has been shown to be reversible in a pancreatic cancer model. Myofibroblastic CAFs are mainly involved in the deposition and reorganization of the extracellular matrix (ECM) in the tumor stroma to induce stiffness and linearized matrix fibrils, which in turn promote tumor growth, cell invasion and metastasis. A stiff ECM can also act as a barrier to prevent therapeutic agent delivery to the tumor and prevent access of immune cells. However, in a different tissue context, CAF-mediated organized ECM can restrain tumor growth and invasion. In parallel of myofibroblast activity, activated fibroblasts secrete different soluble factors (growth factors (GF), cytokines) that can promote tumor growth and invasion, immune suppression by recruiting tumor-associated regulatory T cells (Tregs) and myeloid-derived suppressor cells (MDSCs) and chemoresistance, e.g., via the compensation of signaling pathways and drug efflux

The *fibroblast* is a cell type of paramount importance for extracellular matrix (ECM) production and remodeling in interstitial tissues (Nagalingam et al. 2019). Activated fibroblasts, i.e., *myofibroblasts* and cancer-associated fibroblasts (CAFs), are central in wound healing, tissue fibrosis and tumor fibrosis, and studies of molecular mechanisms have demonstrated that fibroblasts use similar “toolkits” to remodel the ECM in these different conditions (Rybinski et al. 2014; Kalluri 2016; Gullberg et al. 2016). As CAFs have become the subject of more scrutiny in the context of bi-directional tumor stroma interactions, several new functions have been ascribed to them (Fig. 1) (Sahai et al. 2020; Zeltz et al. 2020). A majority of the assays used to probe CAF functions have been performed without considering CAF heterogeneity. With the new knowledge that all tumors contain a tissue-specific repertoire of distinct CAF subtypes, a number of follow-up questions need to be asked: (1) In the case of in vitro cultured CAFs, do the cultured cells represent the in vivo spectrum of subtypes, or has one particular subtype taken over in culture? (2) In the case of immortalized CAFs, which subtypes of CAFs in vivo do they represent? Can results be repeated using primary CAFs? (3) Considering recent findings that the type of mutations in the tumor cells affect the communication with CAFs, should one establish the genetic signature of the cancer cells in tumors from which CAFs were isolated? (4) When novel functions of CAFs are discovered, do they apply to all subtypes of CAFs? Or do these identified new functions pertain to just one CAF subtype and do they depend on the experimental layout? Probably, equally important, in cases where no specific effect on biological function was discovered in a certain CAF assay, was this due to a heterogenous CAF population neutralizing each other’s effects, thus requiring redoing experiments with a focus on CAF subsets? These are all questions that have been generated thanks to new knowledge and that will keep scientists busy for years to come.

## Re-defining fibroblast heterogeneity

In a recent landmark paper Buechler et al*.* have assembled bioinformatic data resulting in an overview of fibroblast heterogeneity, based on transcriptional profiles in normal and “perturbed” mouse tissues as well as in “perturbed” human tissues (Buechler et al. 2021). In the steady-state mouse fibroblast atlas, Buechler and colleagues compiled ten fibroblast clusters of which two represent pan-tissue fibroblasts, distinguished by dominant expression of either Pi16^+^ (peptidase inhibitor 16; a GPI anchored membrane protein) or Col15a1^+^ (collagen XV; a secreted ECM protein) expression, but both also expressing Dpt (dermatopontin; a secreted proteoglycan). The eight remaining clusters were suggested to represent specialized tissue fibroblast subtypes with the following distinguishing upregulated markers: C–C motif chemokine 19 (Ccl19^+^), cochlin (Coch^+^), cartilage oligomeric matrix protein (Comp^+^), C-X-C motif chemokine ligand 12 (Cxcl12^+^), fibulin-1 (Fbln1^+^), bone morphogenetic protein 4 (Bmp4^+^), nephronectin (Npnt^+^) or Hedgehog-interacting protein (Hhip^+^). To create the perturbed mouse fibroblast atlas, the authors integrated 17 scRNA-seq datasets from 13 tissue-associated disease models. Using their approach, they found that the fibroblast population in perturbed conditions can be divided into ten clusters. The universal Pi16^+^ and Col15a1^+^ subsets as well as the Comp^+^, Ccl19^+^, Cxcl12^+^, Hhip^+^ and Npnt^+^ subpopulations were found to be similar to the analogous steady-state fibroblast subtypes. Pi16^+^ fibroblasts were found in perivascular niches and Col15a1 expression was noted deeper inside parenchymal tissues. However, three clusters characterized with dominant expression of leucin-rich repeat-containing protein 15 (Lrrc15^+^), CX-C motif chemokine 5 (Cxcl5^+^) and A disintegrin and metalloprotease like decysin 1 (Adamdec1^+^) appear to represent distinct activated fibroblast populations that are specific to the perturbed-state. Thus, this analysis suggests the existence of universal, tissue and disease specific subpopulations of fibroblasts (Buckley 2021).

Based on these data from Buechler et al*.*, we have analyzed integrin α11 expression in the different clusters. Integrin α11 displayed some low expression on the universal Dpt^+^Pi16^+^ and Dpt^+^Col15a1^+^ fibroblast populations in both normal and perturbed mouse tissues. Buechler et al*.* showed that Dpt^+^Pi16^+^ fibroblasts expressed high levels of genes associated with stemness and predicted that these cells could differentiate into distinct fibroblast populations such as the Lrrc15^+^ cluster, a "perturbation-specific" cluster found in cancer tissues and not present in the steady-state tissues. Interestingly, our analysis of the published dataset indicates the expression of integrin α11 in the Lrrc15^+^ fibroblast subcluster. This transcriptional profile is present in mouse cells from wounds, fibrotic tissues and pancreatic ductal adenocarcinoma (PDAC) and is characterized by α-smooth muscle actin (α-SMA, Acta2) and periostin (Postn) mRNA expression. Buechler et al*.* suggested that these fibroblasts represent a specific myofibroblast population. The expression of integrin α11 mRNA in the Lrrc15^+^ fibroblasts in the assembled dataset was more obvious in the perturbed human tissues. Based on data from three human tissues, the Lrrc15 + myofibroblast-like cluster is found to be enriched with cells from lung- and pancreatic cancers. In contrast, integrin α11 was not highly expressed in the myofibroblast cluster characterized by COL3A1^+^expression and isolated from patients with COVID-19 (Buechler et al. 2021).

The work of Buechler and colleagues, suggesting the existence of universal fibroblast lineages, prompts an update of the current view on the source of activated fibroblasts in different diseases. It will be important to determine how these pan fibroblasts and the different specialized tissue subtypes can be related to the previously described fibroblast subpopulations (discussed briefly in the sections below). Since we observed low mRNA expression of integrin α11 in some universal fibroblast datasets, it will be important to determine the role of α11 in these fibroblast types in the future as well as determining its detailed role in myofibroblast activation in these experimental models.

## Tumor fibrosis

Tumor fibrosis, often termed desmoplasia, is characterized by excessive ECM deposition, ECM reorganization, ECM remodeling and ECM cross-linking, which add up to create a stiff substratum for tumor cells regulating tumor growth, invasion and metastasis (Piersma et al. 2020; Zeltz et al. 2020). Desmoplasia is mediated by subsets of CAFs and is a marker of poor prognosis in several cancer types (Takahashi et al. 2011; Togo et al. 2013; Hao et al. 2019; Rudnick et al. 2021).

Integrins, the transmembrane receptors engaged in cell-ECM and cell–cell interactions, are expressed on CAFs and involved in desmoplasia and matrix remodeling, tumor growth, invasion and chemoresistance (Zeltz et al. 2020). It is important to understand the expression and contribution of integrins in the various steps of tumorigenesis and metastasis for the development of anti-cancer therapies. In this context, we have shown that integrin α11 is overexpressed in the stroma of desmoplastic tumors such as lung, breast and PDAC (Zeltz et al. 2019a). In these cancer types, integrin α11 variably co-localizes with α-SMA expression, suggesting that integrin α11 could be expressed in the subset of CAFs with myofibroblastic features, namely myCAFs (Öhlund et al. 2017). Integrins, however, do not operate in isolation, and tumor growth and invasion depend on integrin crosstalk with growth factor-, chemokine- and non-integrin adhesion receptors, including syndecans (see 3.1 below) expressed on both tumor cells and stromal cells (Desgrosellier and Cheresh 2010; Thomas et al. 2019).

### Integrin-syndecan crosstalk in CAFs

We believe that the different types of interactions between integrins and syndecans have been understudied. The syndecans, a family of four transmembrane heparan sulfate proteoglycans in mammals bind a variety of ligands including ECM components, growth factors, cytokines chemokines, proteases and protease inhibitors. They play major roles in regulating cell signaling, cell–cell and cell-ECM interactions (Couchman et al. 2015). Most published studies on the role of syndecans in cancer have centered changes on the tumor cells, and less attention has been focused on syndecans on CAFs. Loftus et al*.* reported syndecan-2 expression on the cell surface of tumor associated stromal cells within breast tumors. The authors suggest that stromal-derived syndecan-2 influences tumor progression by modulating TGF-β signaling in the tumor microenvironment (Loftus et al. 2021). Stromal syndecan-1 expression in most tumor types is a poor prognostic factor (Couchman 2021). However, the origin of stromal syndecan-1 in carcinomas is mostly unknown and may in some studies been produced by mesenchymal cells or shed from epithelial cells. Both syndecan-1 and syndecan-4 cooperate with integrins and receptor tyrosine kinases to regulate cancer cell-stroma communication (Afratis et al. 2017). Syndecan-1, aberrantly expressed in stromal fibroblasts of invasive breast carcinomas has been proposed to promote fibronectin (FN) assembly through activation of αvβ3 (Yang and Friedl 2016). Studies showed that syndecan-4, in collaboration with integrins, are involved in mechanotransduction and mechanosignaling (Bass et al. 2008; Fiore et al. 2014; Chronopoulos et al. 2020). Chronopoulus et al*.* have demonstrated a force-dependent syndecan-4 and β1 integrin cross-talk. The authors showed that tension on syndecan-4 can initiate a mechanotransduction signaling cascade that led to a synergistic activation of β1 integrins. The activated β1 integrins, in turn, formed new connections to fibronectin, followed by activation of RhoA that induced acto-myosin contraction and increased cellular stiffness (Chronopoulos et al. 2020). In the future, it will be interesting to observe if more integrin-syndecan interactions/cross-talk, will be observed in CAFs.

### CAF and integrin in selected tumor fibrosis

We will next discuss integrin α11 in three types of desmoplastic cancers (lung, breast and pancreatic) and will provide some selected updates on CAFs and CAF-related integrins in each cancer type (Table 1).

**Table 1:** Role of integrin α11β1 and selected CAF integrin-related effectors in desmoplastic tumors

Tumor type	Effector	Experimental model	Role	References
Non-small cell lung carcinoma (NSCLC)	α11β1 integrin	- Itga11^−/−^ SCID mouse model *- *In vitro isolated CAFs from patients	- α11^−/−^ SCID mice exhibit growth inhibition of A549 NSCLC cells and reduced metastasis - α11 regulates expression of LOXL1, a matrix cross-linking enzyme	Navab et al. (2016), Zeltz et al. (2019b)
	Integrin β-like 1 (ITGBL1)	Experimental metastasis mouse model using extracellular vesicles (ECV) from colorectal cancer cells	ITGBL1 in ECV activates lung fibroblasts to establish a premetastatic niche for colon cancer cells	Ji et al. (2020)
Breast cancer	α11β1 integrin	- PyMT//Itga11^−/−^ mouse model *- *In vitro isolated CAFs from mice	- α11^−/−^ PyMT mice display inhibition of breast tumor growth and metastasis - α11^−/−^ CAFs deficient in collagen matrix remodeling	Primac et al. (2019)
	αvβ3 integrin	- In vitro CAFs isolated from mice - In vivo breast cancer mouse models	A novel drug, ProAgio, induces apoptosis in αvβ3 integrin expressing CAF, reducing collagen synthesis, angiogenesis and tumor cell growth	Sharma et al. (2021)
	β4 integrin (ITGB4)	- In vivo co-transplant mouse model - In vitro isolated CAF from patients	ITGB4 induces mitophagy in CAFs to support tumor growth and invasion	Sung et al. (2020)
	Hic-5	In vitro CAFs derived from PyMT mice	- Hic-5 regulates CAF-mediated ECM remodeling via α5β1 integrin - Hic-5 is associated with poor survival related to metastasis	Goreczny et al. (2017, 2018)
	Osteopontin (OPN)	- In vitro primary mouse fibroblasts - In vivo xenograft mouse model	- OPN supports tumorigenesis - OPN stimulates CAF myofibroblastic phenotype to induce EMT in tumor cells	Sharon et al. (2015), Butti et al. (2021)
Pancreatic ductal adenocarcinoma (PDAC)	α11β1 integrin	In vitro cultured Pancreatic stellate cells (PCS)	Knockdown of α11 in PSC inhibits CAF activation and PDAC cell invasion in vitro	Schnittert et al. (2019)
	β5 integrins / iRGD peptide	- In vitro CAFs isolated from PDAC patients - In vivo mouse model	- β5 integrins are induced in PDAC cells in response to CAF-mediated TGF-β secretion - iRGD peptide achieves highly tumor-specific drug delivery in β5 integrins expressing PDAC	Hurtado de Mendoza et al. (2021)
	Matrix stiffness	- In vitro cultured PCS - In vivo xenograft mouse model	Matrix stiffness drives CAF autophagy to create a pro-tumorigenic niche	Hupfer et al. (2021)

*CAF* cancer-associated fibroblast, *ECM* extracellular matrix, *EMT* epithelial-to-mesenchymal transition, *PyMT* polyoma middle T antigen, *SCID* severe combined immunodeficiency

#### Recent developments in lung cancer CAFs

Recently, non-small cell lung carcinoma (NSCLC) CAF heterogeneity has been associated with tyrosine kinase inhibitor (TKI) treatment efficiency (Hu et al. 2021). In this study, the investigators identified three CAF subpopulations based on their expression of hepatocyte growth factor (HGF), fibroblast growth factor-7 (FGF7) and their ability to mediate resistance to osimertinib, a third generation epidermal growth factor receptor (EGFR) TKI. They also showed that TGF-β plays an important role in determining CAF heterogeneity by downregulating HGF and FGF7 expression. In a separate study, Sato et al*.* showed that TGF-β1 released by α-SMA^+^ mouse CAF induced morphological changes and gene expression of the tumor cells, thus may contribute to the histological heterogeneity of lung adenocarcinoma (Sato et al. 2021). With regard to the effect of CAF heterogeneity on the "methylome", Su et al*.* constructed a methylation index (MIND) for NF/CAF discrimination in NSCLC (Su et al. 2021). MIND was based on 54 smoking-associated CpG sites and was suggested to reflect the malignancy of the TME and predict patient survival.

Extracellular vesicles (EC) including exosomes have been suggested to guide metastasis by preconditioning the metastatic niche (Hoshino et al. 2015). In one study, EC vesicles containing integrin β-like1 (ITGBL1) derived from colon cancer cells were reported to reach the lung, where they activated resident fibroblasts to establish a premetastatic niche (Ji et al. 2020). ITGBL1 turns up in many cancer transcriptional profiles. It encodes a secreted protein of 54 kD composed of 10 integrin-like EGF repeats with similarities to the extracellular domain of integrin β1 chain. In one ovarian cancer study, ITGBL1 was shown to associate with β1 integrins and reduce their activity (Cortez et al. 2020). The mode of action for this secreted protein is still unclear, but it might affect the activation status of integrin heterodimers, which might also depend on the nature of the α chain present in the integrin heterodimer with which ITGBL is interacting.

#### Integrin α11 in NSCLC

Our group first showed that integrin α11 among five other genes including COL11A1 (collagen type XI) was differentially expressed in lung adenocarcinoma as compared to normal lung (Wang et al. 2002). On further analyses, the Tsao laboratory analyzed the differentially expressed genes in CAFs versus normal lung fibroblast and identified 22 up-regulated genes in NSCLC CAFs in which ITGA11 and COL11A1 are also present (Navab et al. 2011). The overexpression of integrin α11 introduced it as a novel candidate tumor biomarker in NSCLC. The depletion of integrin α11 in SCID mice inhibits growth of implanted A549 lung adenocarcinoma cells (Navab et al. 2016), indicating that integrin α11 is an important stromal factor in NSCLC. Expression of α-SMA correlated with expression of integrin α11 in this tumor environment, confirming a role of integrin α11 in myofibroblast differentiation also observed in fibrotic tissue. In addition, differential gene analysis of mouse NSCLC stroma revealed the downregulation of Loxl1 in α11^−/−^ mice. Loxl1 is a matrix cross-linking enzyme, which more recently has been shown to be regulated by integrin α11 in stromal cells (Zeltz et al. 2019b). In the NSCLC context integrin α11 is strongly suggested to regulate CAF activation, collagen reorganization and tissue stiffness to promote metastasis (Navab et al. 2016).

**Fig. 2:**
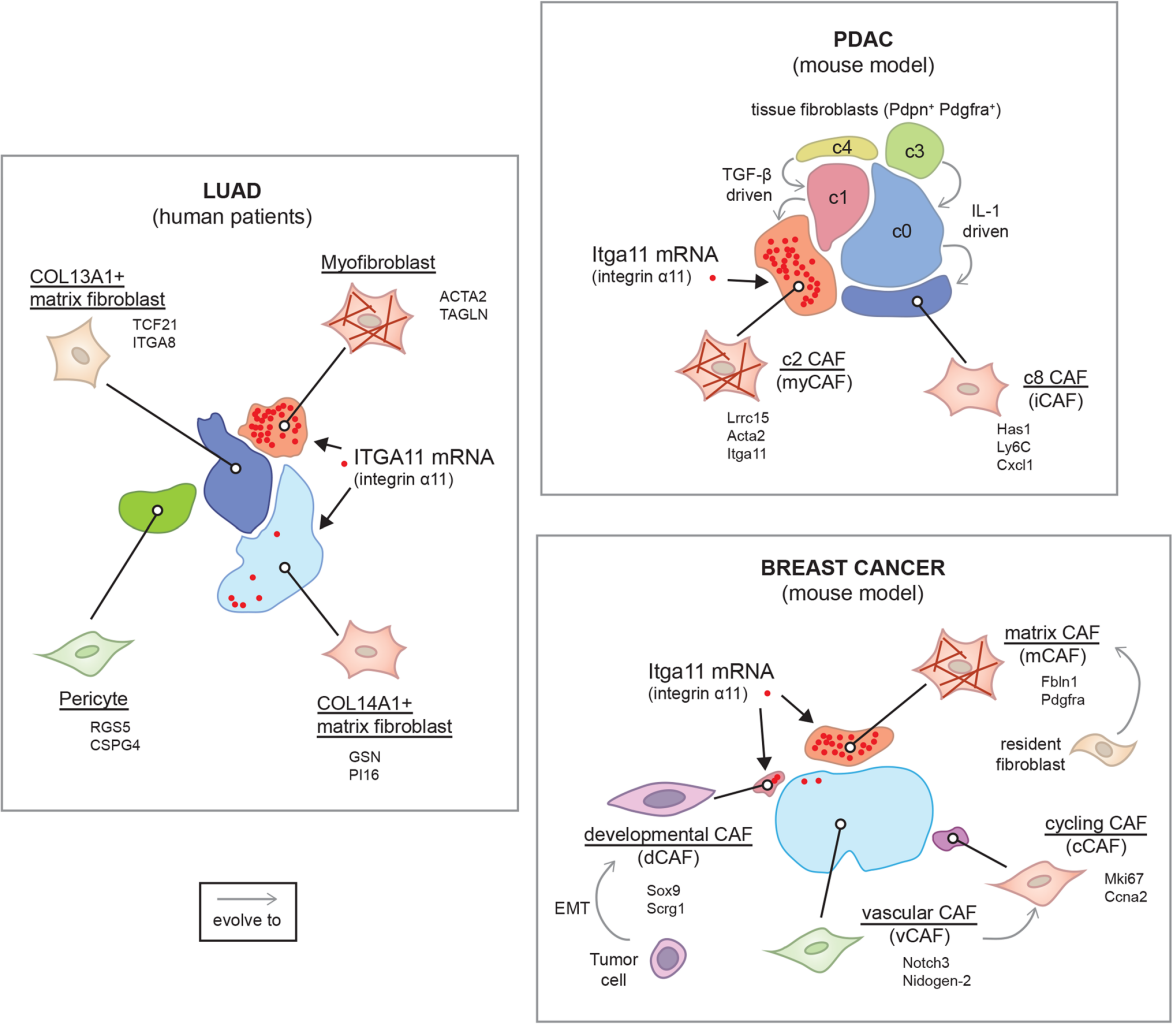
**Integrin α11 expression in CAF heterogeneity**. Main cancer-associated fibroblast (CAF) subpopulations of lung adenocarcinoma (LUAD), pancreatic ductal adenocarcinoma (PDAC) and breast cancer are represented based on scRNA-seq dataset from Kim et al., 2020, Dominguez et al., 2020 and Bartoschek et al., 2018, respectively. For each subset of CAFs, the name is underlined and some specific markers are given. In LUAD, integrin α11 (ITGA11) mRNA is mainly observed in the myofibroblast cluster and in few cells of the COL14A1^+^ matrix fibroblast subset. In the mouse model of PDAC, integrin α11 (Itga11) mRNA is found in the Lrrc15^+^ c2 CAF subpopulation, with myofibroblastic features, which derived from the c1 CAF subset, which itself evolved from the c4 tissue fibroblast population, driven by TGF-β signaling. In parallel, IL-1 drive the evolution of c3 fibroblast subpopulation to c0 and then to the c8 inflammatory CAF subset. In the mouse breast cancer model, integrin α11 (Itga11) mRNA is mostly observed in the matrix CAF subpopulation, thought to derive from resident fibroblasts. Few cells from the “developmental CAF” subset, suggested to be malignant cells that have undergone epithelial-to-mesenchymal transition (EMT), are Itga11^+^

Our analysis of the scRNA-seq dataset of lung adenocarcinoma published by Kim and colleagues (Kim et al. 2020) showed higher expression of integrin α11 mRNA in the myofibroblast cluster that is specific to the tumor environment (Fig. 2). We also observed α11 expression in a subset of Col14A1^+^ matrix fibroblasts, which formed the main subpopulation in normal lung and early-stage tumor. The mechanism contributing to the phenotypic and functional heterogeneity among fibroblasts remains an issue in the lung cancer field. Emerging data on the functional heterogeneity of CAFs in lung cancer have started to appear (Su et al. 2018, 2021; Hao et al. 2019; Hu et al. 2021). Using bulk RNA-seq TCGA data, Dominguez et al*.* described a dominant TGF-β-induced CAF signature of 11 genes (*MMP11*, *COL11A1*, *C1QTNF3*, *CTHRC1*, *COL12A1*, *CL10A1*, *COL5A2*, *THBS2*, *AEBP1*, *LRRC15*, *ITGA11*) in fibroblasts in 31 different advanced human tumors including NSCLS, both LUAD (lung adenocarcinoma) and LUSC (lung squamous cell carcinoma) (Dominguez et al. 2020). In this TGF-β-CAF signature, ITGA11 and COL11A1 are up-regulated in NSCLC compared to normal lung tissue (Wang et al. 2002). Expression of COL11A1 has been reported in gene signatures associated with the TGF-β signaling pathway (Navab et al. 2011; Cheon et al. 2014). COL11A1 has been suggested as biomarker for activated CAFs in epithelial cancer types (Jia et al. 2016). A recent integrated scRNA-seq analysis of CAFs from lung cancer, melanoma, and head and neck squamous cell carcinoma revealed ITGA11 gene expression in another CAF subpopulation called pan-dCAF, with d standing here for desmoplastic (Galbo et al. 2021). Pan-dCAF is a CAF subtype with upregulation of genes coding for various collagens including fibrillar COL1A1 and COL3A1, as well as genes associated with ECM remodeling. Although the pan-dCAF population shared some genes with the above-mentioned TGF-β-CAFs, including *COL11A1, COL12A1*, *CTHRC1* and *THBS2* (thrombospondin-2), it seemed to lack *LRRC15* expression. It should be also noted that the pan-myCAF signature described by Galbo et al. (2021) is characterized by *ACTA2* but does not include *ITGA11* nor *POSTN,* which are both present in the pan-dCAF subset. This difference probably reflects variation in CAF subtypes between different types of cancers as well as differences in experimental design and analysis methods, and also warrants attention when comparing the CAF subtype profiles from distinct studies. The further mapping of the detailed molecular characteristics of CAF subtypes will be needed to stratify the tumors, which will benefit from CAF subtype targeted therapies, and to identify specific CAF subtypes associated with immunotherapy resistance (Grauel et al. 2020).

#### Recent developments in breast cancer CAFs

Cell sorting and scRNA-Seq studies have strived to characterize CAF heterogeneity in primary breast tumors as well as in lymph node and distant metastases and to clarify the functions and molecular mechanisms of these CAF subtypes. Only few examples of these recent investigations are presented here.

Similar to NSCLC and other cancers, one study describes several CAF subtypes in human breast cancer, including ECM-myCAFs and TGF-β-myCAFs, albeit with somewhat different marker gene signatures compared to CAFs from the cancers described above (Kieffer et al. 2020). Interestingly, this work shows signaling loops between ECM-myCAFs, TGF-β-myCAF and Treg lymphocytes leading to the development of an immunosuppressive TME and cancer progression. In another study, the myCAF subset in triple-negative breast cancer (TNBC) was shown to express a similar core mRNA myCAF-signature than described by Dominguez et al. for TGF-β-induced CAFs. These cells were shown to localize in close proximity to invasive tumor cells and to contribute to collagen secretion and alignment (Wu et al. 2020). Mouse models have revealed that expression of the major CAF biomarkers show substantial temporal changes, complicating the comparisons between different studies (Venning et al. 2021).

As a recent example of the functional significance of ECM-related proteins in breast cancer CAFs, siRNA-based knockdown of the endocytic receptor uPARAP (Endo180), which endocytoses both native and denatured collagens, in mouse mammary tumor TGF-β-inducible CAFs, resulted in reduced CAF viability and contraction and limited mammary tumor growth and metastasis (Jungwirth et al. 2021). In another example breast carcinoma cell-secreted matricellular protein osteopontin (OPN) was shown to stimulate CAFs to differentiate into a migratory and collagen-producing myofibroblast tumor-promoting phenotype in a CD44- and-αvβ3-dependent manner (Sharon et al. 2015). In a later study OPN from cancer cells was found to stimulate the CAF myofibroblastic phenotype via ERK and Akt pathways that induced Twist1-dependent gene expression, leading to CXCL12 secretion, which ultimately promoted epithelial-to-mesenchymal transition (EMT) in cancer cells (Butti et al. 2021). OPN is thus an interesting molecule in bi-directional communication between tumor cells and CAFs in breast cancer TME. Both CAFs and endothelial cells express αvβ3 in TNBC. A novel drug, ProAgio, induces apoptosis in an αvβ3-mediated mechanism involving endothelial cells and CAFs (Sharma et al. 2021). Inhibition of αvβ3 on CAFs via ProAgio reduced collagen synthesis and cancer cell proliferation. Inhibition of angiogenesis also reduced the leaky tumor angiogenic vessels and improved drug delivery. The use of this drug is thus an interesting treatment strategy, especially in combination therapies.

Fibronectin and OPN are both present in the breast cancer TME*.* Cancer cells interact via α5β1 with CAF assembled fibronectin (Miyazaki et al. 2020). At the cell surface, integrins assemble into fibrillar adhesions to nucleate fibronectin assembly. When analyzing clinical data, increased levels of the cytosolic protein Hic-5 was observed to worsen diagnosis in basal, HER2+ and grade 3 tumors (Goreczny et al. 2017). Further studies suggested that Hic-5 regulates α5β1 integrin-mediated fibrillar adhesion formation and ECM remodeling in CAFs in a rigidity-dependent fashion, mechanistically suggested to involve a stabilization of the β1 integrin-tensin (a phosphoprotein localizing to integrin-mediated focal adhesions) interaction (Goreczny et al. 2018). The only integrin reported to localize into fibrillar adhesion is α5β1, but it is possible that additional integrins assemble into these matrix-rich structures. In an unconventional exchange of information, exosomes from TNBC cells provide CAFs with the laminin-binding integrin β4 subunit (ITGB4), this induces BNIP3L mitophagy and lactate production, in turn affecting tumor cell proliferation, EMT and invasion (Sung et al. 2020). The authors of this study suggest that targeting ITGB4-induced mitophagy is one possible treatment strategy in breast cancer.

The above investigations and many other recent studies, highlight the complex regulation of breast cancer by CAFs, but also show promising results on therapeutic targeting of CAF components.

#### Integrin α11 in breast cancer CAFs

Bartoschek et al*.* have identified four subpopulations of breast CAFs in a MMTV-PyMT mouse model that they named vascular CAF (vCAF), matrix CAF (mCAF), cell cycle CAF (cCAF) and developmental CAF (dCAF) (Bartoschek et al. 2018). When we analyzed the scRNA-seq data from this study, we observed integrin α11 mRNA expression mainly in the mCAF subclass, which displays the strongest ECM signature and are thought to be derived from resident fibroblasts (Fig. 2). Integrin α11^+^ CAFs are mostly Pdgfrb^+^ and some are Pdgfra^+^ and Acta2^+^. We also noticed some expression of Itga11 mRNA in dCAFs, which harbor expression patterns suggesting that they originate from malignant cells. This is interesting because it suggests that integrin α11 could be expressed on spreading tumor cells that have undergone EMT. In this context, Westcott et al*.* identified ITGA11 as an invasion promoting gene in the leading invasive "trailblazer" cells observed in a breast cancer cell invasion assay in vitro (Westcott et al. 2015).

Wu et al*.* mapped ITGA11 in the myCAF subtype in human TNBC (Wu et al. 2020). We have studied integrin α11 expression in human breast cancer specimens and showed that it is predominantly expressed in tumor stroma in spindle shaped CAFs, sometimes but not always with α-SMA, as well as in α-SMA^+^ ductal myoepithelial cells in in situ cases. High stromal integrin α11 was associated with aggressive subtypes (TNBC, HER2^+^) and high histological grades and proliferation (Smeland et al. 2020).

We also found that in MMTV-PyMT mammary tumor specimens integrin α11 is strongly associated in breast cancer specimens with a PDGFRβ^+^ CAF subset and to lower degrees with PDGFRα, α-SMA, NG2 and FAP (Primac et al. 2019). Like in NSCLC, we could show that depletion of integrin α11 in mice inhibited breast tumor growth and metastasis and that α11^−/−^ CAFs are deficient in remodeling of a collagen matrix. In summary, this study highlighted a cross-talk between α11β1 integrin and PDGFRβ, both receptors interact upon stimulation with PDGF-BB leading to JNK activation and to the deposition of tenascin C, a pro-invasive matricellular protein. We have previously shown that JNK signaling was downstream of the cross-talk between integrin α11 and TGF-βR during collagen reorganization (Schulz et al. 2015) suggesting that in tissue and tumor fibrosis, integrin α11 can cooperate with growth factor receptors and signal through JNK to regulate the ECM. Thus, targeting JNK might be an alternative option to block the profibrotic activities of integrin α11.

#### Recent developments in pancreatic cancer CAFs

In most models of tumor stroma interactions, a majority of published data suggests that the tumor stroma is tumor supportive (Han et al. 2015; Alexander and Cukierman 2016). In molecular terms this corresponds to various types of cross-talk between tumor and stromal cells including paracrine- as well as integrin-mediated signaling. In pancreatic cancer, the stroma has been suggested to support tumor growth, tumor metastasis and to be involved in tumor chemoresistance (Pan et al. 2015). Other studies suggest that stroma might act as a restraining barrier preventing tumor expansion and tumor spread. With the increased awareness about CAF heterogeneity within TME many published studies might have to be revisited and the effects of TME re-examined in more detail, keeping in mind the CAF heterogeneity. New data generated in more targeted approaches to CAF subsets support data from widely cited paper from Özdemir et al*.* suggesting that conditional deletion of α-SMA-expressing fibroblasts in experimental PDAC mouse model worsened tumor outcome (Özdemir et al. 2014). Experimentally the α-SMA-thymidine kinase mouse were crossed with two different models of PDAC, namely the LSLS-^KrasG12D/+^;Trp53^R172H/+^;Pdx ^cre/+^ (KPC) mouse and the Ptf1a^cre/+^; Kras^Gt2D/+^;TGFbr2 ^flox/flox^ (PKT) mouse, and cell depletion of α-SMA expressing cells was induced with ganciclovir. These rather drastic cell depletion protocols with reduced number of myofibroblasts resulted in more invasive, undifferentiated, and necrotic tumors. The cell population targeted in this approach were most likely the CAFs we now call myCAFs. In a more recent publication, an advanced PDAC mouse model was used to analyze the effects of collagen I deletion in α-SMA-expressing cells and in the context of TME (Chen et al. 2021). The results indicate that deletion of collagen I, makes tumor more proliferative, supporting the concept that the collagen I- producing myCAFs are really tumor re-restraining. Interestingly, detailed analysis of the PDAC tumor model by Chen et al*.* suggest that myCAFs lacking collagen I synthesis stimulate the PDAC cells to secrete CXCL5 which in turn attracts neutrophils contributing to an immunosuppressive environment, which further explains this interesting phenotype. These results are in agreement with recent data from a study of tumor collagen content arguing for a correlation between degree of desmoplasia and disease severity (Jiang et al. 2020). PDAC patients with more desmoplasia had a better prognosis which led the authors to inhibit LOXL2 activity in a mouse tumor model. Their data thus also support the concept that a collagen matrix restrains PDAC tumor growth, while this effect is tumor- and context -dependent. In this background, the question is to determine what is the role of integrin α11, whether it is supporting or restraining the pancreatic tumor. The effect of stiffness in PDAC has been studied in vitro using pancreatic tumor cell lines and pancreatic stellate cells (PSCs). This data demonstrates that changing stiffness, metabolically reprograms stromal cells to undergo autophagy in an αv integrin- and FAK-dependent manner, creating a pro-proliferative niche supporting cancer cell growth. Mechanistically this is brought about by post-translational stabilization of AMP-activated kinase (Hupfer et al. 2021). In PDAC models, CAFs have in a mechanism involving TGF-β been demonstrated to upregulate αvβ5 levels in PDAC cells, in turn making them an efficient drug target for the tumor-penetrating iRGD peptide (Hurtado de Mendoza et al. 2021).

#### Integrin α11 in pancreatic cancer

In breast and lung cancer, we previously mentioned that integrin α11 is expressed in myofibroblastic CAFs and contributes to tumorigenicity. When it comes to its expression in the collagen-rich PDAC stroma, integrin α11 mRNA has been identified in a LRRC15^+^ CAF subpopulation in PDAC, that is derived from TGF-β-activated CAFs, characterized as myCAFs (Dominguez et al. 2020) (Fig. 2). Using the selected monoclonal antibody mAb 203E3, we have found that integrin α11 protein was restricted to the stromal compartment of PDAC and was localized in PDGFRβ^+^, α-SMA^+^ and/or FAP^+^ CAF, but not in NG2^+^ CAFs (Zeltz et al. 2019a). The origin of integrin α11-expressing CAFs in PDAC is still uncertain, i.e., whether they are derived from stellate cells or from resident fibroblasts. However, the lack of NG2 colocalization would suggest that α11 expressing CAFs are not pericyte-derived. It is interesting to note that α11β1 is upregulated in human PSC activated with PDAC conditioned media in vitro (Schnittert et al. 2019). Similar to other cancer models, abrogation of integrin α11 in the stromal cells in vitro reduced CAF activation and matrix reorganization. Furthermore, in this study knockdown of integrin α11 reduced paracrine secretion from stellate cells, inhibiting PDAC cell invasion (in vitro). Our previous studies using α11 mAbs need to be repeated with more markers to conclude if α11 is expressed on stellate cell-derived CAFs in vivo and to sort out the origin and role of PDAC α11^+^ CAFs in PDAC.

Integrin α11 mRNA has also been shown to be strongly expressed in desmoplastic liver, ovary, uterus and head and neck carcinoma where it colocalizes with α-SMA (Parajuli et al. 2017; Zeltz et al. 2019a). The TME is quite complex and CAF activation might depend on the tumor type and mutations that can modify how tumor cells and CAFs interact. myCAFs in different tumor types may share matrix production and remodeling features, but still express tissue specific markers and mechanisms of induction. Although integrin α11 seems to be strongly associated with myCAF activation independent of the desmoplastic features, not all α11^+^ CAFs are α-SMA^+^, suggesting that myofibroblast differentiation in vivo might only occur in specific CAF subsets. Further studies are indeed required to better characterize the phenotype of CAFs that express integrin α11 and thus determining the function of this collagen-binding integrin in each CAF subpopulation.

## Integrin α11 tools for tumor fibrosis studies and therapeutics

### Monoclonal antibodies directed against human integrin α11

We have generated integrin α11 monoclonal antibodies (mAbs) for two different purposes: for tissue detection and for use as blocking antibodies. Integrin α11 is barely detectable in human adult normal tissues; however, it is upregulated in tumor fibrosis, implicating integrin α11 as a potential biomarker that can predict patient outcome. In order to specifically detect integrin α11 in different fibrotic tissues, we used mAb 203E3 in cryosection analysis of α11 expression (Zeltz et al. 2019a) and mAb 210F4 for paraffin-embedded tissues (Smeland et al. 2020). These immunostaining-designed antibodies will be useful to confirm expression of integrin α11 in the different fibroblast subpopulations that we have mentioned. In this review, we have described the pro-fibrotic functions of integrin α11 in the tumor tissues studied, suggesting that integrin α11 is an interesting target for anti-fibrosis therapy. One candidate for combination treatment and antibody drug conjugate-based treatments is the α11 mAb 203E1 which we have shown can interfere with cell-collagen interactions (Zeltz et al. 2019a).

### ITGA11-driver Cre mouse strain

scRNA-seq data have shown that fibroblasts involved in fibrosis are heterogeneous, but lack of fibroblast-specific markers is an important issue when studying the role of these fibroblasts. To study in more details the role of genes in α11-expressing fibroblasts, we have generated an ITGA11-driver Cre-recombinase mouse strain (Alam et al. 2020). We have shown that during wound healing and in fibrotic heart, ITGA11-driven Cre expression is induced. ITGA11-driven Cre was expressed in 60% of isolated mouse embryonic fibroblasts, indicating that integrin α11 is only expressed in a subset of these fibroblasts. Thus, ITGA11-Cre mouse strain is a useful tool for cell lineage tracing and for gene deletion in subpopulations of fibroblast. It could also be an attractive tool to delete cells in an α11-specific manner if crossed with ROSA26iDTR mice (Buch et al. 2005) to determine function of fibroblast subsets in tumor fibrosis.

## Conclusion

Expression of integrin α11 is heterogeneous among mesenchymal cells. In tumor fibrosis, integrin α11 is mainly found in myofibroblast subpopulations, which are associated with TGF-β signaling and matrix reorganization, where it may mediate myofibroblast differentiation and collagen remodeling. However, we also observed integrin α11 expression in some other fibroblast clusters in fibrotic tissues that needs to be confirmed and in which its role remains to be defined. For this purpose, we have generated new integrin α11 tools that will be helpful in further studies of the pro-fibrotic integrin α11.

### scRNA-seq data used for integrin α11 expression

*Buechler *et al*.*, R Seurat objects of mouse perturbed-state atlas and human perturbed-state atlas; *Kim *et al., interactive lung cancer cell atlas (URECA); *Bartoschek *et al*.*, R scripts of scRNA-seq of breast CAF.
